# Tapping into Metabolomics for Understanding Host and Rotavirus Group A Interactome

**DOI:** 10.3390/life15050765

**Published:** 2025-05-10

**Authors:** Phiona Moloi Mametja, Mmei Cheryl Motshudi, Clarissa Marcelle Naidoo, Kebareng Rakau, Luyanda Mapaseka Seheri, Nqobile Monate Mkolo

**Affiliations:** 1 Department of Biology and Environmental Sciences, Sefako Makgatho Health Sciences University, Pretoria 0204, South Africa; phiona.phiolocity@gmail.com (P.M.M.); cheryl.motshudi@smu.ac.za (M.C.M.); clarissa.naidoo@smu.ac.za (C.M.N.); 2 Diarrheal Pathogens Research Unit, Department of Virology, Sefako Makgatho Health Sciences University, Pretoria 0204, South Africa; kebareng.rakau@smu.ac.za (K.R.); mapaseka.seheri@smu.ac.za (L.M.S.)

**Keywords:** omics, butyrate, histo-blood group antigens, rotavirus P[8] strain, children

## Abstract

Group A rotavirus continues to be a leading global etiological agent of severe gastroenteritis in young children under 5 years of age. The replication of this virus in the host is associated with the occurrence of Lewis antigens and the secretor condition. Moreover, histo-blood group antigens (HBGAs) act as attachment factors to the outer viral protein of VP4 for rotavirus. Therefore, in this study, we employed a metabolomic approach to reveal potential signature metabolic molecules and metabolic pathways specific to rotavirus P[8] strain infection (VP4 genotype), which is associated with the expression of HBGA combined secretor and Lewis (Le) phenotypes, specifically secretor/Le^(a+b+)^. Further integration of the achieved metabolomics results with lipidomic and proteomics metadata analyses was performed. Saliva samples were collected from children diagnosed as negative or positive for rotavirus P[8] strain infection (VP4 genotype), which is associated with the HBGA combined secretor/Le^(a+b+)^. A total of 22 signature metabolic molecules that were downregulated include butyrate, putrescine, lactic acid, and 7 analytes. The upregulated metabolic molecule was 2,3-Butanediol. Significant pathway alterations were also specifically observed in various metabolism processes, including galactose and butanoate metabolisms. Butyrate played a significant role in viral infection and was revealed to exhibit different reactions with glycerolipids, glycerophospholipids, sphingolipids, sterol lipids, and fatty acyls. Moreover, butyrate might interact with protein receptors of free fatty acid receptor 2 (FFAR2) and free fatty acid receptor 3 (FFAR3). The revealed metabolic pathways and molecule might provide fundamental insight into the status of rotavirus P[8] strain infection for monitoring its effects on humans.

## 1. Introduction

One of the primary causes of illness and mortality in children under the age of five, particularly in low-income countries, is known as diarrheal illness [1]. Before the 1970s, the cause of infantile gastroenteritis in many cases remained unknown, but a breakthrough was made in 1973 when viral particles were found in samples from duodenal biopsies taken from children who had severe diarrhea [2]. The newly found virus was given the name rotavirus, derived from the Latin word rota, which means wheel, due to its distinctive circular morphological form [3]. Diarrheagenic viruses of rotavirus fall under the genus *Rotaviruses* in the Sedoreoviridae family (formerly Reoviridae), with at least eight rotavirus species, *Rotavirus A* to *Rotavirus H*, classified [3,4]. They are further classified into genotypes utilizing nucleotide sequences of the inner protein shell of the virus protein (VP) 7 (G) and an outer capsid of VP4 (P) [5]. Group A rotaviruses are non-enveloped double-stranded RNA (dsRNA) viruses that have a complex architecture of three concentric capsids that surround a genome of 11 segments of dsRNA [6]. The RNA segments encode structural viral proteins (VP1, VP2, VP3, VP4, VP6, and VP7) that prompt faster viral maturity and therefore increase host specificity, cell entry, and enzymatic functions necessary to produce viral transcripts, and they contain epitopes that generate immune responses [7]. Among them, VP7 G1-G4, G9, and G12, integrated with VP4 P[4], P[6], and P[8], are implicated in severe cases of human rotavirus diarrhea, which are commonly observed globally [8]. The P[8] genotype of rotavirus is the most common cause of infections worldwide [9,10,11,12]. Moreover, groups of fucose-containing carbohydrates known as histo-blood group antigens (HBGAs) have been associated with the attachment of rotavirus as an attachment factor or receptor [13]. These antigens are also distributed in biological fluids including saliva, gastric fluid, milk, and blood content as free oligosaccharides [14,15]. The HBGAs are governed by several genes with silent alleles, which consist of Lewis antigens, secretors, and blood phenotypes A, B, and O (ABOs) [14,16]. Phenotypes of Lewis antigen profiles include non-secretors Le^(a+b−)^, secretors Le^(a−b+)^, and secretors Le^(a+b+)^ or Le^(a−b−)^ that might be non-secretors or secretors [17].

Group A rotavirus is still leading globally as a viral etiological agent of severe gastroenteritis in young children under 5 years of age. Even after the introduction of rotavirus vaccines, Rotarix^®^ (GSK Biologicals, Rixensart, Belgium) and RotaTeq^®^ (Merck & Co., White River, PA, USA), in the year 2009 by the World Health Organization (WHO), rotavirus is still responsible for a yearly projection of 128,500 deaths worldwide [18]. Nonetheless, detailed research on virus–host interactomes has provided us with noteworthy insights pertaining to how host features contribute to infection and the virus’s replication inside the host. The advent of technology-based approaches to omics has surfaced as a promising direction for further understanding of the pathogenesis of the viruses and for revealing proteins, RNAs, and metabolites of hosts associated with virus infection, besides producing possibilities for productive therapeutic interventions [19,20]. Despite the challenges we face with group A rotavirus, there is a lack of insight into the application of omics technologies to investigate the virus–host interactions of rotavirus. This highlights the necessity of employing the spatial technology-based approaches of omics to identify viral infection biomarkers of metabolic molecules and metabolic pathways. This approach is closely associated with disease or infection phenotypes, since it can reflect downstream effects of the responsible lipids and proteins. Thus, the aim of this study was to integrate a metabolomics approach with lipidomic and proteomics metadata analyses to reveal potential signature metabolic molecules and metabolic pathways specific to rotavirus P[8] strain infection (VP4 genotype), which is associated with the expression of HBGA combined secretor and Lewis (Le) phenotypes, specifically secretor/Le^(a+b+)^.

## 2. Materials and Method

### 2.1. Ethical Approval

The Sefako Makgatho Health Sciences University Research and Ethics Committee approved this study (Ethics reference no: SMUREC/S/254/2023:PG). Parents or guardians provided consent for sample collections from children under 12 months of age.

### 2.2. Sample Population and Collection

The South African national surveillance program currently monitors rotavirus and related etiologies of severe diarrhea in children who are under treatment at different health care facilities. Eligible children were recruited according to the WHO generic protocol, which is regulated for hospital-based rotavirus surveillance, as described by Mwenda et al. [21] and Rakau et al. [22]. The recruited children were attending Oukasie Primary Health Care Clinic and formed part of the ongoing diarrheal surveillance in the Diarrheal Pathogens Research Unit founded at Sefako Makgatho Health Sciences University and Dr. George Mukhari Academic Hospital. Oukasie Primary Health Care Clinic is one of the allied health clinics of Dr. George Mukhari Academic Hospital.

As previously recorded by Rakau et al. [22], as part of surveillance, a series of saliva samples were collected from children at different time points (<4 weeks, 10 weeks, 14 weeks, and 12 months). At the age of 12 months, the male children had acute watery diarrhea, and some were diagnosed negative (H type 1-; *n* = 5) and positive (H type 1+; *n* = 5) for rotavirus P[8] strain infection (VP4 genotype) associated with the expression of histo-blood group antigen (HBGA) combined secretor and Lewis (Le) phenotypes, specifically secretor/Le^(a+b+)^. These children diagnosed as negative or positive for rotavirus had received two doses of Rotarix vaccination at 6 and 14 weeks. Saliva collection was carried out using a plain sterile oral swab (AEC-Amersham Soc. Ltd., Johannesburg, South Africa) by pressing the swab on the surface of the left and right cheeks and under the tongue for approximately 10 s. Subsequently, the swabs were squeezed through a syringe immediately after collection, and the expressed saliva was kept at −20 °C in a freezer in 4 mL cryovials (Carl Roth GmbH & Co., Karlsruhe, Germany). Subsequently, the samples were transported (1.4 km) from Dr. George Mukhari Academic Hospital to the Diarrheal Pathogens Research Unit, a WHO Rotavirus Regional Reference Laboratory. Upon arrival, the saliva samples were directly stored at −80 °C at the Diarrheal Pathogens Research Unit for further analysis.

### 2.3. Metabolic Molecular Profiling

#### 2.3.1. Reagents and Chemicals

N, O-Bis(trimethylsilyl)trifluoroacetamide with 1% trimethylchlorosilane (BSTFA containing 1% TMCS) was purchased from Sigma-Aldrich (St. Louis, MO, USA). The utilized organic solvents were purchased from ultra-pure Burdick and Jackson (Honeywell International Inc., Muskegon, MI, USA).

#### 2.3.2. Preparation of Samples

The sample preparation procedure was adapted from Mueller et al. [23]. Saliva samples were thawed at room temperature, and 50 μL of 3-Phenylbutyric acid (100 ppm) and 25 μL of Heptadecanoic acid (not used for quantitative purposes; test extraction efficiency) were added to 50 μL of each sample. Some of the two samples were less than 50 µL; in this case, the available volumes of 40 µL for both samples were aliquoted into a microcentrifuge tube to achieve reproducible results as well as to reduce the impact of pre-analytical variations. For protein precipitation, 500 μL of methanol/acetonitrile (70/30 *v*/*v*) was added, thoroughly vortexed, and kept at −20 °C for 1 h. The samples were then centrifuged at 4000 rpm (790× *g*) at 4 °C for 15 min, and the supernatant was transferred to a glass vial. A second extraction was conducted by adding 100 μL of methanol/water (8:1 *v*/*v*) to the residue. The mixture was vortexed, kept for 20 min at −20 °C, and then centrifuged for 15 min at 4000 rpm at 4 °C. The organic phases were combined and evaporated until dry under a gentle stream of nitrogen. For derivatization purposes, 50 μL of the MOX solution (20 mg/mL) in pyridine was added and incubated for 30 min at 60 °C. Afterward, 50 μL of BSTFA containing 1% TMCS was added and incubated for 30 min at 60 °C. It was, thereafter, transferred to a glass insert and injected into a Two-Dimensional Gas Chromatography system (GCxGC).

#### 2.3.3. Two-Dimensional Gas Chromatography with Time-of-Flight Mass Spectrometer System

The system utilized was a Pegasus 4D GCxGC-TOFMS (Leco Corporation, St. Joseph, MI, USA), operating an Agilent 7890A GC (Agilent, Atlanta, GA, USA) linked to a time-of-flight mass spectrometer (TOFMS) (Leco Corporation, St. Joseph, MI, USA) supplied with a Gerstel Multi-Purpose Sampler (MPS) (Gerstel GmbH & Co. KG, Eberhard-Gerstel-Platz 1, Mülheim an der Ruhr, Germany). A cryogenic cooler was also installed in the system. The primary GC oven temperature was initially programmed at 70 °C for 2 min, whereafter it increased at 4 °C/min to 300 °C, where it was kept for 2 min. The programming of the modular was set at a temperature of 100 °C for 2 min, and the temperature was subsequently increased to 310 °C for 12 min. The programming of the secondary GC oven was set at a temperature of 85 °C for 2 min; thereafter, the temperature was increased to 300 °C for 5 min. The effluent that appeared from the primary column onto the secondary column was controlled using cryomodulation and a hot nitrogen gas pulse that persisted for 0.3 s every 3 s.

#### 2.3.4. Data Processing for Peak Identification

Leco Corporation ChromaTOF software (version 4.71) was used for peak identification and mass spectral deconvolution at an S/N ratio of 200, requiring at least 3 apexing peaks. High-resolution chromatography coupled with a fast acquisition and non-scanning mass spectrometer allowed accurate deconvolution. Mass fragmentation patterns were produced by the MS, and according to their particular GC retention times, the identification of the names of the peaks was performed by comparing them to commercially obtainable NIST spectral libraries (mainlib, replib.) with at least 700 (70%) resemblance. Identification of small peaks at level 3 was accomplished, as mentioned by Schymanski et al. [24], by tentative candidate structures based on partial spectral similarities or diagnostic ions.

### 2.4. Integration of Metabolomics with Lipidomic and Proteomics Metadata Analyses

Through metabolomics analysis in this study, the revealed potential high-signature metabolic molecule, butyrate, which might play a significant role in rotavirus P[8] strain infection that is associated with the HBGA combined secretor/Le^(a+b+)^, was further subjected to metadata analysis. Metadata analysis was performed to reveal lipids and proteins that interact with butyrate. The Lipid Maps, www.lipidmaps.org (accessed on 22 August 2024), database was used to identify human lipids that react with butyrate, while the SwissLipids, https://www.swisslipids.org/ (accessed on 22 August 2024), database was utilized to reveal human proteins that interact with butyrate. The three-dimensional structure of the revealed human proteins that interact with butyrate was studied using a template-free machine learning algorithm, P2Rank, which was integrated into PrankWeb (https://prankweb.cz/ (accessed on 22 August 2024)). The pockets were visualized based on the number of amino acid residues, probability of binding, average AlphaFold score, and average conservation score. PaxDb, the Protein Abundance Database (version 5.0), https://pax-db.org/compute (accessed on 22 August 2024), was utilized to assess normal human salivary abundance, while the STRING database, https://string-db.org/ (accessed on 22 August 2024), was utilized for the determination of the protein–protein interaction network of the revealed human proteins that interact with butyrate.

### 2.5. Data Analysis

GraphPad Prism version 8.2.0 (GraphPad Software, Inc., San Diego, CA, USA) was utilized for statistical analysis, and all samples were run in five replicates. Multivariate and univariate normalized data analyses were conducted to analyze all-encompassing untargeted-omics data using Metaboanalyst version 6.0 https://www.metaboanalyst.ca (accessed on 24 April 2025). Univariate data analyses were conducted to reveal signature metabolic molecules (carbohydrates, peptides, lipids, nucleotides, amino acids, and organic acids) that meet the criteria of *p*-value < 0.05, fold change (FC) > 2.0, and variable importance in projection (VIP) > 1.5. The Benjamini–Hochberg false discovery rate (FDR) was used to perform multiple comparison correction to reduce false positives (FDR-corrected *p*-values (*q*-values) < 0.05). Supervised approaches were employed to visualize and classify small potential signature molecules using partial least squares discriminant analysis (PLS-DA), orthogonal partial least squares discriminant analysis (OPLS-DA), and sparse partial least squares discriminant analysis (sPLS-DA). The predictive ability (Q2) and fitness (R2) of the PLS-DA and OPLS-DA models were also evaluated. Pathway and enrichment analyses were performed using the Kyoto Encyclopedia of Genes and Genome (KEGG) *Homo sapiens* (human) database by means of global test algorithms.

## 3. Results

### 3.1. Saliva Quantification

**Figure 1:**
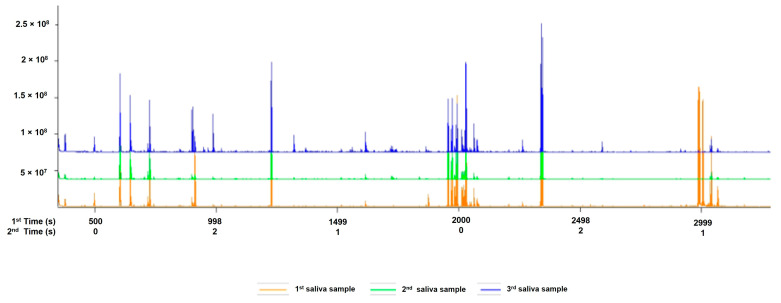
A typical example of the total ion chromatogram (TIC) peaks of saliva metabolic molecules from infected and uninfected children.

The saliva samples comprised 155 total ion chromatogram (TIC) peaks (Figure 1, Table S1, Supplementary Material). A total of 120 peaks were identified, and 35 peaks were unidentified.

### 3.2. Univariate and Chemometrics Analysis

**Figure 2:**
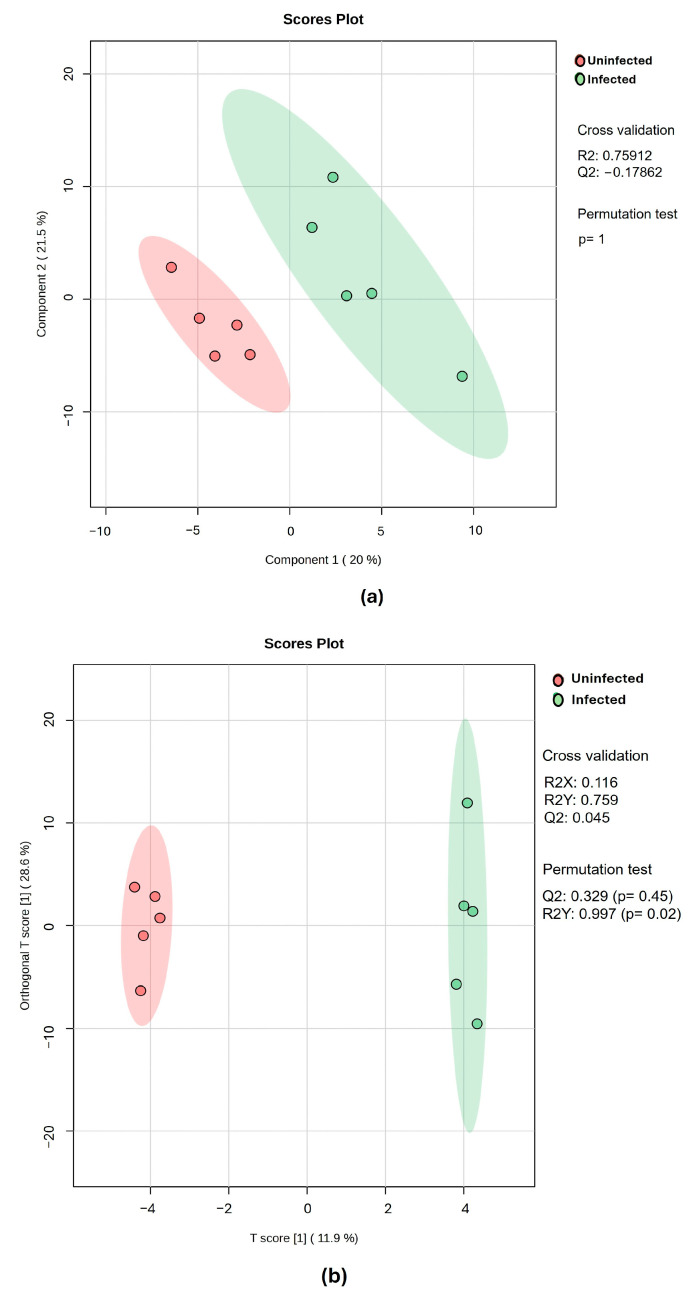
The score scatter plots of the (**a**) PLS-DA and (**b**) OPLS-DA models of metabolic molecules from children’s saliva samples. Saliva samples from uninfected and infected children with the rotavirus P[8] strain associated with HBGA combined secretor and Lewis phenotypes (secretor/Le^(a+b+)^).

**Figure 3:**
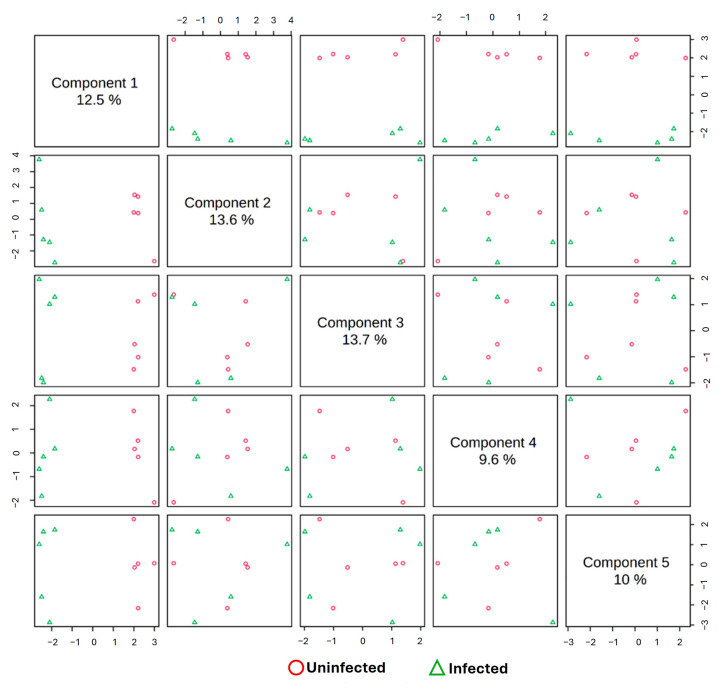
The sparse PLS-DA pairwise score plot for the top 5 components of metabolic molecules from children’s saliva samples. Saliva samples from uninfected and infected children with the rotavirus P[8] strain associated with HBGA combined secretor and Lewis phenotypes (secretor/Le^(a+b+)^).

Score plots of the PLS-DA, OPLS-DA, and sparse PLS-DA models were utilized for categorization and visualization of metabolic molecule variations that can lead to a decrease in non-specific effects and prediction of the potential signature saliva metabolic molecules of rotavirus P[8] strain infection associated with HBGA combined secretor and Lewis phenotypes (secretor/Le^(a+b+)^). As depicted in Figure 2, there was a notable segregation pattern between the children’s saliva samples with rotavirus and those without rotavirus at 12 months. The predictive ability and fitness of the PLS-DA and OPLS-DA models are depicted in Figure 2 and Figures S1 and S2 of Supplementary Material. A clear and concise segregation summary of the sparse PLS-DA algorithm pairwise score plot for the top five components (variations of 20%, 13.5%, 13.7%, 9.6%, and 10% for each component) between the saliva samples of the rotavirus-infected and uninfected children is depicted in Figure 3.

**Figure 4:**
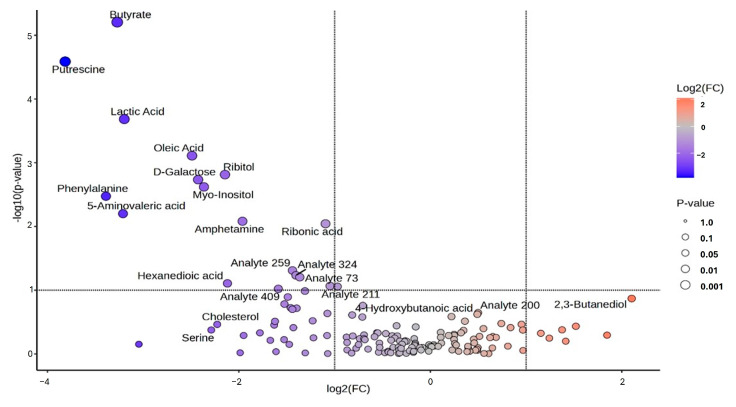
Volcano plot analysis of changes in metabolic molecules from children’s saliva samples uninfected and infected with rotavirus-P[8] associated with HBGA combined secretor and Lewis phenotypes (secretor/Le^(a+b+)^).

**Figure 5:**
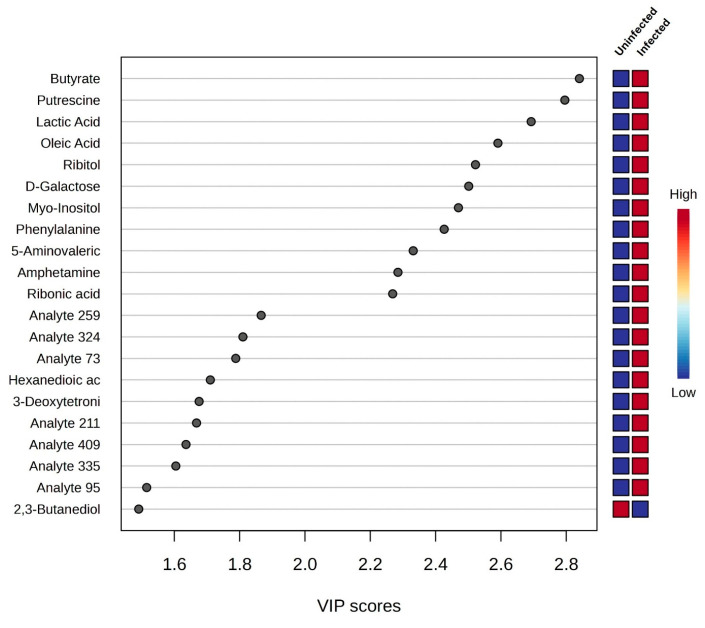
Variable importance in projection (VIP > 1.5) score plot identified by PLS-DA displays the potential signature metabolic molecules from saliva samples of children uninfected and infected with the rotavirus-P[8] strain associated with HBGA combined secretor and Lewis phenotypes (secretor/Le^(a+b+)^). The colored boxes on the right signify the relative concentrations of the related metabolic molecules from uninfected and infected children with the rotavirus P[8] strain.

Univariate analysis was employed whereby the volcano plot was constructed using fold change (FC) values (>2.0) and the *t*-test (*p* < 0.05) as the selection criteria for identifying the degree of metabolic molecule difference, as shown in Figure 4. A total of 106 metabolic molecules were not significant, while 43 were downregulated and 7 were upregulated (Figure 4). Among the 22 metabolic molecules with a VIP greater than 1.5, those that were downregulated include butyrate, putrescine, lactic acid, oleic acid, ribitol, D-galactose, myo-inositol, phenylalanine, 5-aminovaleric acid, amphetamine, ribonic acid, hexanedioic acid, 3-deoxytetronic acid, 2,3-butanediol, and 7 analytes (Figure 5, Table 1). The upregulated metabolic molecule with a VIP greater than 1.5 was 2,3-Butanediol (Figure 5, Table 1). However, after false discovery rate (FDR) correction, butyrate, putrescine, lactic acid, oleic acid, ribitol, D-Galactose, myo-Inositol, phenylalanine, 5-Aminovaleric acid, amphetamine, ribonic acid, and analyte-259 were the metabolites discovered to have a significant association with rotavirus infection with the Benjamini–Hochberg-adjusted *p*-value range of ≤0.016 (Table 1).

### 3.3. The Kyoto Encyclopedia of Genes and Genome Pathway

**Figure 6:**
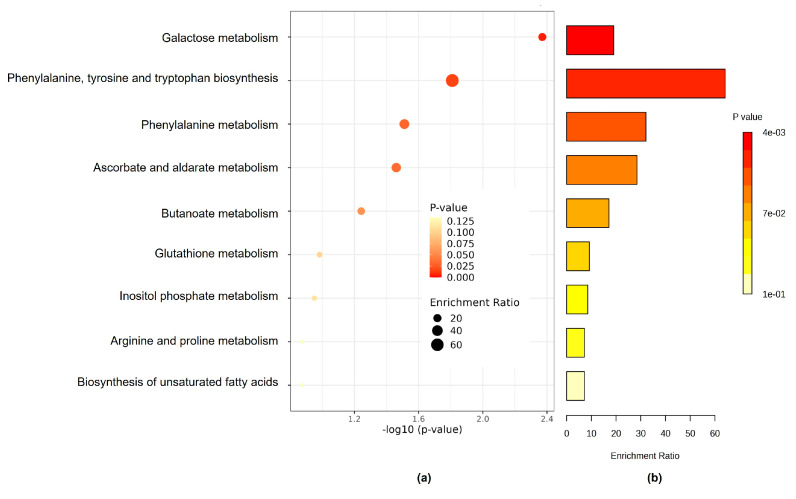
The KEGG pathway of the significantly different metabolic molecules between uninfected and infected children with the rotavirus P[8] strain associated with the HBGA combined secretor/Le^(a+b+)^. (**a**) Overview of enriched metabolic molecule sets; (**b**) visualized enrichment ratio.

**Figure 7:**
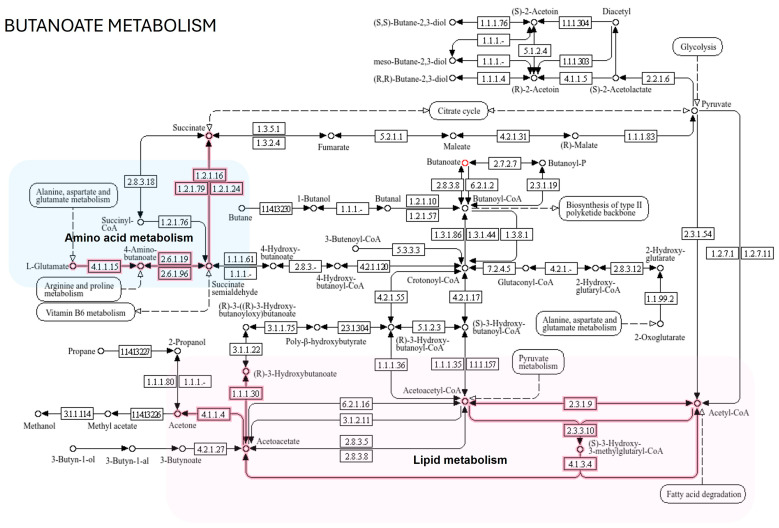
The KEGG pathway shows butanoate metabolism of butyrate, a potential high-signature metabolic molecule between uninfected and infected children with the rotavirus P[8] strain associated with HBGA combined secretor/Le^(a+b+)^. Solid black arrows represent molecular interaction. The dotted black arrows indicate indirect link of metabolites with metabolic pathway. Red lines represent metabolites associated with amino acid metabolism (highlited in blue) and lipid metabolism (highlited in light pink).

Different Kyoto Encyclopedia of Genes and Genome pathways (KEGG) and results from the global test for enrichment analysis related to rotavirus P[8] strain infection—which is associated with the expression of HBGA combined secretor and Lewis (Le) phenotypes, specifically secretor/Le^(a+b+)^—were identified, as shown in Figure 6 and Table 2. To determine the distinct saliva metabolic pathways associated, the conducted test used *p* < 0.05 and Impact > 0.1 as screening parameters. The prominent pathways are galactose metabolism; phenylalanine, tyrosine, and tryptophan biosynthesis; phenylalanine metabolism; ascorbate and aldarate metabolism; butanoate metabolism; glutathione metabolism; inositol phosphate metabolism; biosynthesis of unsaturated fatty acids; and arginine and proline metabolism. Galactose metabolism and butanoate metabolism (p < 0.05 and Impact > 0.1) are the key metabolic pathways that reflect the rotavirus P[8] strain infection that is associated with the HBGA combined secretor/Le^(a+b+)^ (Table 2). The revealed potential high-signature metabolic molecule, butyrate, is mainly associated with butanoate metabolism, and it is involved in amino acid metabolism and lipid metabolism, as depicted in Figure 7.

### 3.4. Integration of Metabolomics with Lipidomic and Proteomics Metadata Analysis

**Figure 8:**
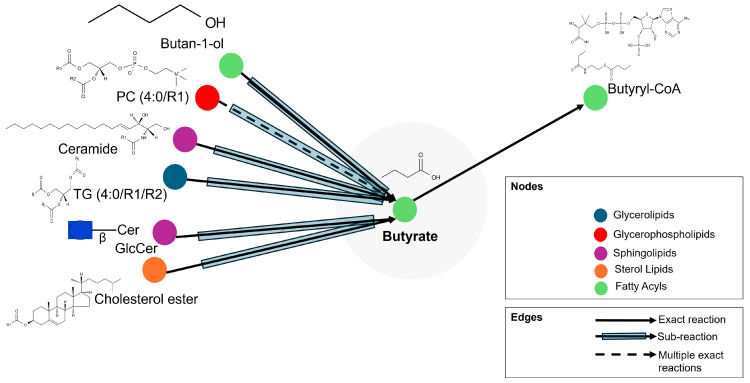
Diagrammatical representation of butyrate exact, sub-, and multiple exact reactions with glycerolipids, glycerophospholipids, sphingolipids, sterol lipids, and fatty acyls.

As mentioned in the previous section, metabolomics analysis revealed butyrate as a potential high-signature metabolic molecule that played a significant role in rotavirus P[8] strain infection that is linked to the HBGA combined secretor/Le^(a+b+)^. Butyrate exhibits exact, sub-, and multiple exact reactions with glycerolipids, glycerophospholipids, sphingolipids, sterol lipids, and fatty acyls, as depicted in Figure 8. Butyrate exhibits exact reactions and sub-reactions with fatty acyls of butyryl-CoA and butan-1-ol. Moreover, butyrate also exhibits sub-reactions with sphingolipids (ceramide, GlcCer), glycerolipid (TG (4:0/R1/R2)), and sterol lipid (cholesterol ester). Multiple exact reactions of glycerophospholipid (PC (4:0/R1)) are also exhibited with butyrate.

**Figure 9:**
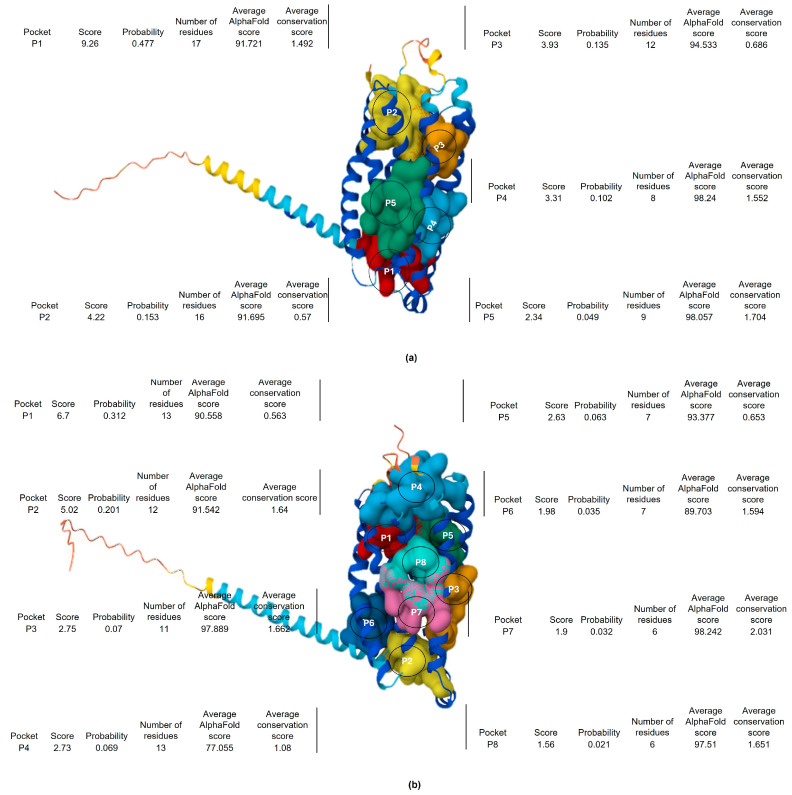
Structural representation of human (**a**) free fatty acid receptor 2 (FFAR2) and (**b**) free fatty acid receptor 3 (FFAR3) proteins that interact with butyrate. The diagram depicts pockets visualized based on the number of amino acid residues, probability of binding, average AlphaFold score, and average conservation score. Key: ^P1–P8^ pockets 1 to 8.

**Figure 10:**
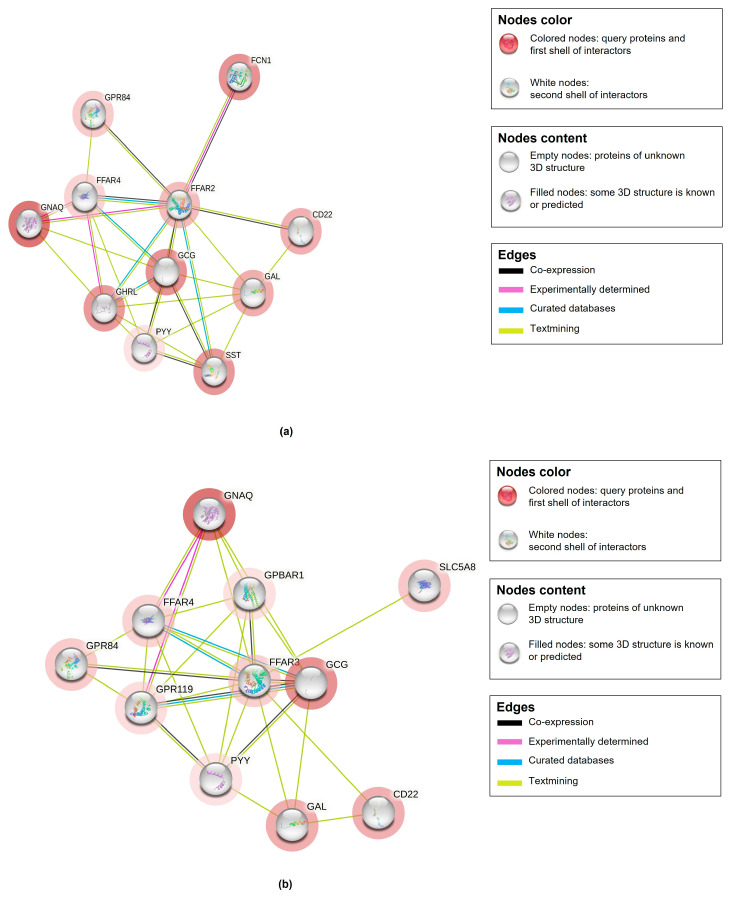
Protein–protein interaction network functional enrichment analysis of human (**a**) free fatty acid receptor 2 (FFAR2) and (**b**) free fatty acid receptor 3 (FFAR3) proteins that also interact with butyrate.

The observed proteins that interact with butyrate are free fatty acid receptor 2 (FFAR2) and free fatty acid receptor 3 (FFAR3) proteins. Proteins of FFAR2 and FFAR3 are equivalently distributed in the saliva-secreting glands with an interaction consistency score of 30.1, coverage of 54%, and abundance levels of 10,486 ppm (Table 3). Figure 9 depicts the 3D structure of human FFAR2 and FFAR3 proteins that interact with butyrate. Amino acid residues of FFAR2 have five pockets with probability scores of 0.477, 0.153, 0.135, 0.102, and 0.049 for each pocket 1 to 5, and they exhibited an average conservation score of 1.492, 0.57, 0.686, 1.552, and 1.704, respectively. On the other hand, amino acid residues of FFAR3 have eight pockets with probability scores of 0.312, 0.201, 0.07, 0.069, 0.063, 0.035, and 0.032 for each pocket 1 to 8, and they exhibited an average conservation score of 0.563, 1.64, 1.662, 1.08, 0.653, 1.594, 2.031, and 1.651, respectively. The closer the probability score is to 1, the greater the likelihood that the predicted pocket is an actual ligand binding site. Conservation scores of amino acids on a scale fluctuating from 0 to 9 represent conserved amino acids. Moreover, pockets of FFAR2 and FFAR3 proteins varied in terms of the number of amino acids, as depicted in Figure 9. Protein–protein interaction network analysis of FFAR2 and FFAR3 revealed 28 and 27 interactions (edges), with each consisting of 10 proteins (nodes), respectively (Table 3, Figure 10). The featured proteins that interact with FFAR2 proteins are guanine nucleotide-binding protein G(q) subunit alpha (GNAQ), free fatty acid receptor 4 (FFAR4), peptide YY (PYY), appetite-regulating hormone (GHRL), ficolin-1 (FCN1), G protein-coupled receptor 84 (GPR84), cluster of differentiation-22 of B-cell receptor (CD22), galanin peptides (GAL), glucagon (GCG), and somatostatin (SST). The featured proteins that interact with FFAR3 proteins are free fatty acid receptor 4 (FFAR4), peptide YY (PYY), G protein-coupled receptor 84 (GPR84), B-cell receptor CD22 (CD22), galanin peptides (GAL), glucagon (GCG), glucose-dependent insulinotropic receptor (GPR119), G-protein coupled bile acid receptor 1 (GPBAR1), sodium-coupled monocarboxylate transporter 1 (SLC5A8), and guanine nucleotide-binding protein G(q) subunit alpha (GNAQ).

## 4. Discussion

It is well understood that several viruses utilize different approaches to complete their replication by capturing the host’s metabolism [25]. In omics approaches, for instance, metabolomics analysis is an optimal technique for the identification of metabolic molecules dedicated to virus infection. For example, in the study of Fontaine et al. [26], during dengue virus infection, central carbon metabolism, specifically glycolysis, was significantly upregulated. Metabolomic analysis offers valuable insights into biochemical actions that signal the phenotype or cellular state in different biological contexts, including the saliva sample [27,28]. Besides the fact that salivary glands may act as reservoirs for rotavirus [14], saliva can provide insights into systemic metabolic alterations influenced by gut microbiota and gut dysbiosis. These alterations can be utilized to discover possible biomarkers for gut-associated diseases [28,29,30]. Therefore, we herein employed a spatial technology-based omics approach to reveal potential signature metabolic molecules that can be dedicated to rotavirus P[8] strain infection, which is also associated with the expression of HBGA combined secretor and Lewis (Le), specifically secretor/Le^(a+b+)^. We further integrated the achieved metabolomics results with lipidomic and proteomics metadata analyses.

Total ion chromatogram peaks of 155 metabolites were discovered from salivary samples of children diagnosed as rotavirus-infected and uninfected. Cross-validation and permutation tests did not support the robustness of the model PLS-DA, due to the model being trained on a small dataset, leading to overfitting [31], although the R2 value indicated a better fit. In conformity with Kjeldahl and Bro [32], the PLS-DA model is susceptible to overfitting. Nevertheless, permutation tests supported the robustness of the OPLS-DA score plot model with an observation of two distinct clusters. We revealed signature metabolic molecules that met the criteria of *p*-value < 0.05, FC > 2.0, and VIP > 1.5. Moreover, the FDR-corrected *p*-value < 0.05 (*q*-values) was also estimated. Applying these criteria and cut-offs to the datasets used in this study improved the power of the analysis by directing it to metabolic molecules with strong evidence of difference and predictive power. In terms of VIP values, the greater-than-one rule is typically used to identify descriptors with the highest importance in the projection [33]. Student’s *t*-test (*p*-value < 0.05) was used to determine which metabolites could discriminate across groups in the dataset [34]. Studies have also revealed that instrument variability is smaller than biological variability for mammals, which implies that fold change thresholds of 1.5–2.0 must be utilized [35]. The analysis revealed potential signature metabolic molecules which were downregulated, including butyrate, putrescine, lactic acid, oleic acid, ribitol, D-galactose, myo-inositol, phenylalanine, 5-aminovaleric acid, amphetamine, ribonic acid, hexanedioic acid, 3-deoxytetronic acid, 2,3-butanediol, and seven analytes. The upregulated metabolic molecule was 2,3-Butanediol. Moreover, after FDR correction was applied, these metabolites were also discovered to have a significant association with rotavirus infection, except for Hexanedioic acid, 3-Deoxytetronic acid, 2,3-Butanediol, and six analytes. These significant metabolites belong to different classes such as organonitrogen, hydroxy acid, fatty acyls, organooxygen, carboxylic acid, and benzene. The class with the greatest alteration is fatty acyls. Some of these different classes have also been identified in studies on dengue virus [26,36]; however, to our knowledge, their associated potential metabolic molecules specific to rotavirus P[8] strain infection, which can be linked to the HBGA combined secretor/Le^(a+b+)^, have not been explored.

Among the revealed potential signature metabolic molecules, butyrate showed a significant association with rotavirus P[8] strain infection that is linked to the HBGA combined secretor/Le^(a+b+)^. Butyrate is a short-chain fatty acid produced in the colon of mammalians by bacterial fermentation of carbohydrates [37,38]. Butyrate has several significant biological functions and binds to various receptors. In humans, butyrate is among the two primary endogenous agonists of hydroxycarboxylic acid receptor 2 (HCA2), which is a G protein-coupled receptor [39]. Butyrate is necessary for homeostasis of the host’s immunity [40]. Butyrate has been shown to be a critical mediator of the colonic inflammatory response. Fecal samples of patients with inflammatory bowel diseases, including both Crohn’s disease and ulcerative colitis, contained butyrate that was downregulated [36,41,42]. In this study, we discovered that butyrate levels from saliva samples were also significantly downregulated. The observed association of downregulated butyrate, which might have an influence on rotavirus replication, is supported by an in vitro study by Zhao et al. [43], where sodium butyrate exerted defensive effects against rotavirus-induced cell apoptosis through inhibiting endoplasmic reticulum stress-mediated apoptosis by regulating the phosphorylated protein kinase-like endoplasmic reticulum kinase and eukaryotic initiation factor 2 alpha signaling pathway via GPR109A. In a similar study, sodium butyrate reduced the oxidative stress triggered by rotavirus infection and restored intestinal mucosal mechanical barrier function by activation of the adenosine monophosphate-activated protein kin signal pathway mediated by the receptor GPR109A [44]. The downregulation of phenylalanine, putrescine, myo-inositol, oleic acid, lactic acid, 5-aminovaleric acid, 3-deoxytetronic acid, and 2,3-butanediol has also been implicated mainly in inflammatory bowel diseases, including both Crohn’s disease and ulcerative colitis [36,45,46,47,48,49]. However, hexanedioic acid, ribonic acid, and D-galactose have not yet been implicated in any inflammatory bowel diseases. Hexanedioic acid has mainly been implicated in 3-hydroxy-3-methylglutaryl-CoA lyase deficiency, malonyl-CoA decarboxylase deficiency, carnitine–acylcarnitine translocase deficiency, and medium-chain acyl-CoA dehydrogenase [50,51]. Ribonic acid signals the incomplete metabolism of several metabolic molecules [52], with D-galactose being produced through a buildup of D-galactose-1-phosphate as a result of enzyme GALT deficiency in humans [53]. However, the upregulated 2,3-Butanediol in this study is conceded as a microbial metabolite formed from pyruvate through various intermediates comprising diacetyl α-acetolactate and acetoin [54].

The Kyoto Encyclopedia of Genes and Genome pathway analysis provides insight into different ways of rotavirus infection, which might alter the metabolic pathways of the host cells [55], and as a result, ways of treating rotavirus infections can be identified. Through these analyses, we observed multiple pathways associated with rotavirus P[8] strain infection. These altered pathways include galactose metabolism; phenylalanine, tyrosine, and tryptophan biosynthesis; phenylalanine metabolism; ascorbate and aldarate metabolism; butanoate metabolism; glutathione metabolism; inositol phosphate metabolism; biosynthesis of unsaturated fatty acids; and arginine and proline metabolism. Significant alterations were also specifically observed mainly in galactose metabolism when compared to other altered pathways. Rotavirus binds to cellular receptors of glycans formed by monosaccharides, for example, galactose and glucose [56]. The O-glycan biosynthesis pathway can enable viral attachment and replication by altering the composition and functionality of the host cell glycocalyx [57]. Galectins, a family of carbohydrate-binding proteins, play a role in the Leloir pathway, which is a galactose-related pathway, through interactions with glycans and binding to β-galactose-containing glycoconjugates, leading to rotavirus attachment and replication [58,59]. Galactose can be a derivative of the breaking down of lactose [60], and rotavirus infection can decrease the production of the enzyme lactase, which is responsible for the digestion of lactose, leading to diarrhea [61]. Rotavirus infection can also modify the energy metabolism of host intestinal cells, resulting in higher levels of lactate formation, lower levels of mitochondrial oxygen utilization, and diminished cellular adenosine triphosphate [62]. In a recent study by Mitra et al. [55], the nucleotide metabolism pathway (pyrimidine and purine) of the citric acid cycle and the alanine–aspartate–glutamate pathway were associated with rotavirus HT-29 cell infection.

The revealed potential high-signature metabolic molecule of butyrate is mainly associated with butanoate metabolism, and it is involved in amino acid metabolism and lipid metabolism. The integrated lipidomic metadata analyses revealed that butyrate is involved in exact, sub-, and multiple exact reactions with glycerolipids, glycerophospholipids, sphingolipids, sterol lipids, and fatty acyls. The implicated lipids are butyryl-CoA, butan-1-o, ceramide, GlcCer, TG (4:0/R1/R2)), cholesterol ester, and PC (4:0/R1). In the study by Gaunt et al. [63], concentrations of cholesterol ester and ceramide lipids were upregulated in cells infected with rotavirus. Lipids play several roles, as energy sources, membrane components, and signaling molecules, due to their structural variety of approximately 40,000 species [64]. The structural diversity of these lipids has been implicated in the alteration of receptor agonist or antagonist actions [65,66]. The action of lipids, for instance, cholesterol, is among the main factors facilitating host and rotavirus interactions on the virus surface of host pattern recognition receptors [67]. Rotavirus is predicted by pattern recognition receptors including melanoma differentiation-associated gene 5 (MDA-5), Toll-like receptor-3 (TLR-3), and retinoic acid-induced gene-1 (RIG-1) [68]. In this study, proteomic metadata analyses identified protein receptors that interact with butyrate to be FFAR2 and FFAR3. These cell-surface receptors from the G-protein-coupled receptor family sense short-chain fatty acids from the gut microbiota. They bind to butyrate, a short-chain fatty acid, and together they regulate different physiological functions, which include energy, immune response, metabolism, and inflammation [69]. Their capabilities in cell signaling might play a role in human rotavirus infection [69], as they are expressed in rotavirus-targeted tissues, particularly within the intestinal epithelium, and they influence immune responses through the action of short-chain fatty acids produced by the gut microbiota [69,70,71]. However, their direct involvement in rotavirus entry is not well established [71,72]. Therefore, this outcome, based on the structural importance, activation, and expression pattern of FFAR2 and FFAR3 receptors by metabolites of the gut microbiota at varying tissue levels, may prompt new research directions for investigating their roles in human diseases, which includes human rotavirus infections. However, this investigation contains limits that must be considered. The small sample size and lack of more diverse cohorts may limit the findings’ generalizability and reliability. The restricted sample size may weaken the statistical power, leading to false negatives, and the lack of diverse cohorts may restrict the ability to transfer findings to diverse populations [73]. Consequently, our research emphasizes the necessity of using a larger sample size and more diverse cohorts in future studies to increase the generalizability and validity of the findings for the enhancement of biomarker discovery. Moreover, unidentified metabolites were found that hindered the ability to fully interpret their role in biological processes. While the non-significant findings of some metabolites after FDR correction provided valuable insights, further investigation is warranted.

## 5. Conclusions

The present study reveals multiple pathways and different metabolic molecules associated with rotavirus P[8] strain infection. Butyrate was identified as a potential signature metabolic molecule that is significantly dedicated to rotavirus P[8] strain infection, which is also associated with the expression of HBGA combined secretor and Lewis (Le) phenotypes, specifically secretor/Le^(a+b+)^. Butyrate might exhibit exact, sub-, and multiple exact reactions with glycerolipids, glycerophospholipids, sphingolipids, sterol lipids, and fatty acyls. In addition, butyrate may interact with the protein receptors of FFAR2 and FFAR3. Therefore, the discovered metabolic pathways and molecules might provide fundamental insight into the status of rotavirus P[8] strain infection for monitoring its effects on humans.

## Figures and Tables

**Table 1:** Identified potential signature metabolic molecules from saliva samples of children infected and uninfected with rotavirus-P[8].

Metabolic Molecules	Molecular Weight (g/mol)	Formula	Class	VIP	FC	*p*-Value Fisher’s Exact Test	FDR Critical Value	FDR *q*-Value
Butyrate	88.1051	C_4_H_8_O_2_	Fatty acyls	2.793	0.104	0.000	0.002	0.000 ††
Putrescine	88.1515	C_4_H_12_N_2_	Organonitrogen compound	2.749	0.071	0.000	0.005	0.000 ††
Lactic acid	90.0779	C_3_H_6_O_3_	Hydroxy acid	2.648	0.109	0.000	0.007	0.000 ††
Oleic acid	282.4614	C_18_H_34_O_2_	Fatty acyls	2.548	0.178	0.001	0.010	0.005 ††
Ribitol	152.1458	C_5_H_12_O_5_	Organooxygen compound	2.480	0.226	0.002	0.012	0.005 ††
D-Galactose	180.1559	C_6_H_12_O_6_	Organooxygen compound	2.460	0.186	0.002	0.014	0.005 ††
Myo-Inositol	180.16	C_6_H_12_O	Organooxygen compound	2.429	0.194	0.002	0.017	0.005 ††
Phenylalanine	165.1891	C_9_H_11_NO_2_	Carboxylic acid	2.386	0.096	0.003	0.019	0.005 ††
5-Aminovaleric acid	117.1463	C_5_H_11_NO_2_	Carboxylic acid	2.293	0.108	0.006	0.021	0.007 ††
Amphetamine	135.2062	C_9_H_13_N	Benzene	2.247	0.257	0.008	0.024	0.013 ††
Ribonic acid	166.1293	C_5_H_10_O_6_	Organooxygen compound	2.231	0.468	0.009	0.026	0.015 ††
Analyte 259 UM159	***	***	***	1.835	0.369	0.049	0.029	0.016 ††
Analyte 324 UM73	***	***	***	1.780	0.378	0.058	0.031	0.079 *†
Analyte 73 UM116	***	***	***	1.758	0.389	0.063	0.033	0.087 *†
Hexanedioic acid	146.1412	C_6_H_10_O_4_	Fatty acyls	1.682	0.230	0.078	0.036	0.088 *†
3-Deoxytetronic acid	120.1039	C_4_H_8_O_4_	Hydroxy acid	1.648	0.484	0.086	0.038	0.091 *†
Analyte 211 UM110	***	***	***	1.640	0.484	0.094	0.040	0.095 *†
Analyte 409 UM256	***	***	***	1.608	0.333	0.095	0.043	0.095 *†
Analyte 335 UM74	***	***	***	1.578	0.403	0.093	0.045	0.095 *†
Analyte 95 UM168	***	***	***	1.490	0.356	0.069	0.048	0.095 *†
2,3-Butanediol	90.121	C_4_H_10_O_2_	Organooxygen compound	1.466	4.297	0.002	0.050	0.095 *†

Key: VIP: variable importance in projection; UM: unique mass; FC: fold change; *** unknown; FDR: false discovery rate; †† significant; *† not significant.

**Table 2:** Metabolomic pathway analyses of saliva from rotavirus-infected and uninfected children.

Pathway Name	Hits^a^	*p-*Value	Holm *p*^b^	Impact Value
Galactose metabolism	2	0.00426	0.341	0.105
Phenylalanine, tyrosine, and tryptophan biosynthesis	1	0.0155	1	0.0156
Phenylalanine metabolism	1	0.0308	1	0.0312
Ascorbate and aldarate metabolism	1	0.0346	1	0.0351
Butanoate metabolism	1	0.0542	1	0.0585
Glutathione metabolism	1	0.104	1	0.109
Inositol phosphate metabolism	1	0.112	1	0.117
Arginine and proline metabolism	1	0.133	1	0.14
Biosynthesis of unsaturated fatty acids	1	0.133	1	0.14

Hits^a^ represents the number of metabolites in one pathway. Holm *p*^b^ indicates the statistical *p*-values that were further adjusted using the Holm–Bonferroni method for multiple tests.

**Table 3:** Humans’ saliva-secreting gland abundances and protein–protein interaction network analysis of FFAR2 and FFAR3 proteins that interact with butyrate.

	Saliva-Secreting Glands Abundance in *Homo sapiens* *			Protein–Protein Interaction Network **					
Proteins Interacting with Butyrate ***	Interaction Consistency Score	Coverage	Abundance Levels (ppm)	No. of Nodes	No. of Edges	Avg. Local Clustering Coefficient	Avg. Node Degree	Exp. No. of Edges	PPI Enrichment *p*-Value
Free fatty acid receptor 2	30.1	54%	10,486	11	28	0.835	5.09	11	9.72 × 10^−6^
Free fatty acid receptor 3	30.1	54%	10,486	10	27	5.4	0.837	9	1.25 × 10^−6^

Source: * PaxDb: Protein Abundance Database (version 5.0) https://pax-db.org/compute (accessed on 22 August 2024); ** STRING database https://string-db.org/ (accessed on 22 August 2024); *** SwissLipids database https://www.swisslipids.org/ (accessed on 22 August 2024); Key: Avg: Average; PPI: protein–protein interaction; Exp.: expected.

## Data Availability

Data are included in the article or Supplementary Materials; further inquiries can be directed to the corresponding author.

## Supplementary Materials

The following supporting information can be downloaded at https://www.mdpi.com/article/10.3390/life15050765/s1. Table S1: GCxGC-TOFMS revealed metabolites from saliva samples of rotavirus-infected and uninfected children (12 months age); Figure S1: PLS-DA classification using different number of components. (**a**) Cross-validation test (**b**) Permutation test. The red star indicates the best classifier. Figure S2: OPLS-DA classification using different number of components. (**a**) Cross-validation test (**b**) Permutation test.
