# Supplementary material for: Tapping into Metabolomics for Understanding Host and Rotavirus Group A Interactome
Source: Life (Basel). 2025 May 10;15(5):765. doi: 10.3390/life15050765 (PMC12113392; doi:10.3390/life15050765)
Supplement: Supplementary file 1 [file life-15-00765-s001.zip › Figure S1 S2 supplimentry material.pdf]

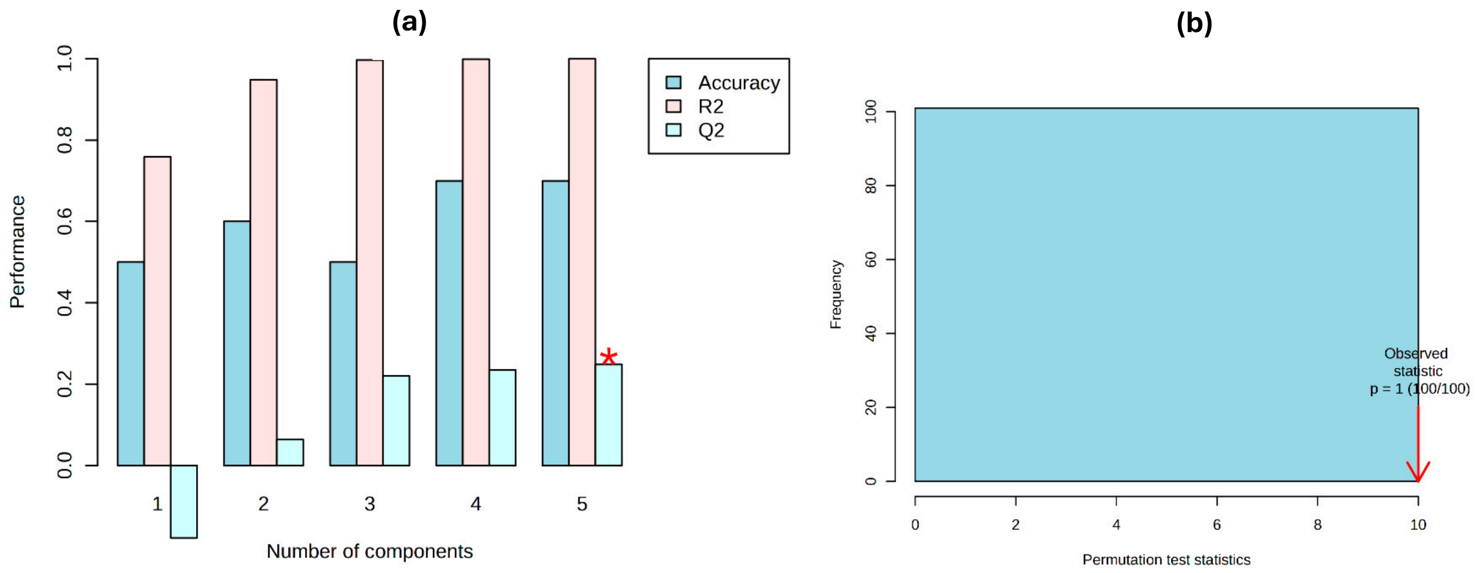

Figure S1: PLS-DA classification using different number of components. (**a**) Cross-validation test (**b**) Permutation test. The red star indicates the best classifier.

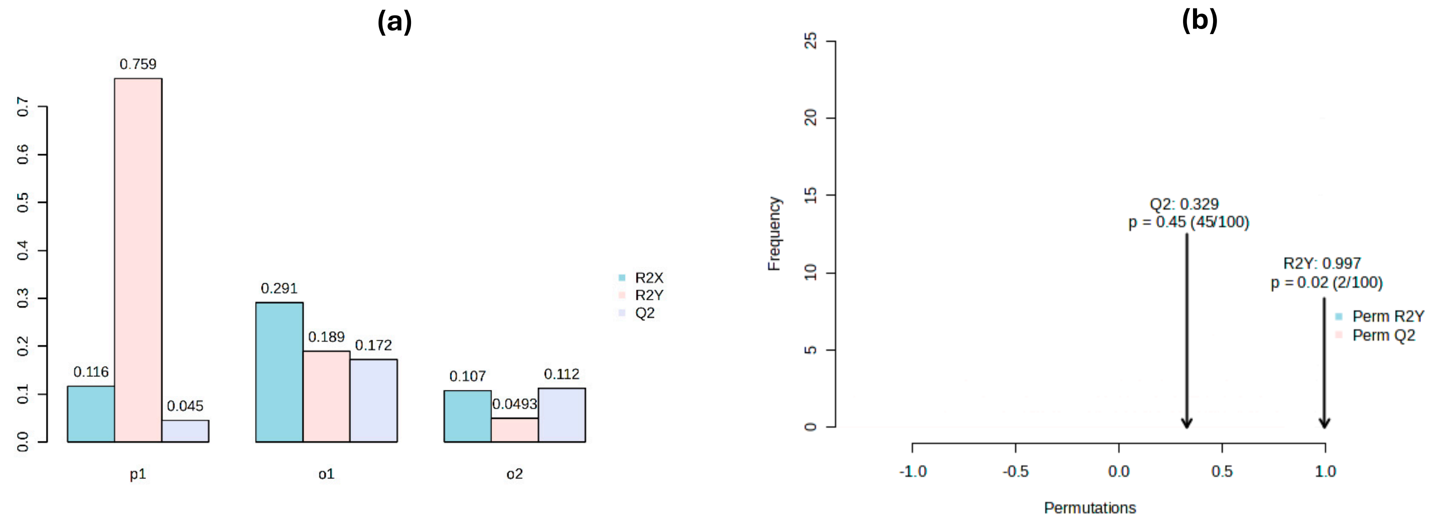

Figure S2: OPLS-DA classification using different number of components. (**a**) Cross-validation test (**b**) Permutation test.
